# Relationship Between Bone Metabolic Markers and Presence of Sarcopenia in Patients with Type 2 Diabetes Mellitus: A Cross-Sectional Study

**DOI:** 10.3390/jcm14175973

**Published:** 2025-08-24

**Authors:** Tomoyuki Matsuyama, Yoshitaka Hashimoto, Noriyuki Kitagawa, Takafumi Osaka, Masahide Hamaguchi, Michiaki Fukui

**Affiliations:** 1 Department of Endocrinology and Metabolism, Graduate School of Medical Science, Kyoto Prefectural University of Medicine, 465 Kajii-cho, Kawaramachi-Hirokoji, Kamigyo-ku, Kyoto 602-8566, Japan; tomo10@koto.kpu-m.ac.jp (T.M.); nori-kgw@koto.kpu-m.ac.jp (N.K.); tak-1314@koto.kpu-m.ac.jp (T.O.); mhama@koto.kpu-m.ac.jp (M.H.); michiaki@koto.kpu-m.ac.jp (M.F.); 2 Department of Diabetes and Endocrinology, Matsushita Memorial Hospital, 5-55 Sotojimacho, Osaka 570-8540, Japan; 3 Department of Diabetology, Kameoka Municipal Hospital, 1-1 Shinonoda, Shino-cho, Kyoto 621-8585, Japan; 4 Department of Endocrinology and Diabetology, Ayabe City Hospital, 20-1 Otsuka, Aono-cho, Kyoto 623-0011, Japan

**Keywords:** sarcopenia, osteoporosis, diabetes

## Abstract

**Objectives**: We investigated the relationship between bone metabolic markers or bone mineral density (BMD) and sarcopenia in patients with type 2 diabetes mellitus (T2DM). **Methods**: In this cross-sectional study involving 119 subjects (76 women and 43 men), bone metabolic markers were evaluated by bone alkaline phosphatase and bone tartrate-resistant acid phosphatase (TRACP-5b). BMD was measured using the dual-energy X-ray absorptiometry method, and sarcopenia was diagnosed using skeletal muscle mass index (SMI), evaluated by body composition measurement and handgrip strength. **Results**: Significant correlation was observed between handgrip strength or SMI and TRACP-5b in both sexes (correlation coefficients were −0.50 in handgrip strength and −0.41 in SMI in men; −0.25 in handgrip strength and −0.21 in SMI in women). Furthermore, significant correlation was observed between handgrip strength or SMI and BMD of the femoral neck in both sexes (correlation coefficients were 0.33 in handgrip strength and 0.44 in SMI in men; 0.34 in handgrip strength and 0.47 in SMI in women). The concentrations of TRACP-5b with sarcopenia were significantly higher than those without (643.8 ± 261.9 vs. 455.7 ± 165.6 mU/dL), and BMD of femoral neck with sarcopenia was significantly lower than those without (0.54 ± 0.12 vs. 0.66 ± 0.16 g/cm^2^). TRACP-5b (odds ratio 1.05, 95% confidence interval 1.01–1.10) and femoral neck BMD (odds ratio 0.30, 95% confidence interval 0.14–0.68) were associated with the presence of sarcopenia after adjustment for confounders. **Conclusions**: TRACP-5b and BMD of the femoral neck were associated with sarcopenia in patients with T2DM.

## 1. Introduction

In Japan, where the population is rapidly aging, maintaining bone and muscle health is important for healthy life expectancy [1]. A strong correlation between bone density and muscle mass has been reported [2,3,4], and osteoporosis and sarcopenia have attracted attention.

Osteoporosis is a condition characterized by chronically reduced bone strength leading to fractures. Bone strength is composed of two factors: bone density and bone quality [5]. It has been reported that elderly patients with femoral neck fractures have a 5-year survival rate of approximately 50%, a worse prognosis than gastric cancer [6,7], whereas treatment of osteoporosis has been reported to improve survival [8]. Reducing the prevalence of osteoporosis and increasing treatment rates are important to support medical care for older people [8]. Diabetes is recognized as a condition that not only affects bone density but also has a significant impact on bone quality [5]. There is a consensus that diabetes mellitus causes bone fragility, and indeed, patients with poorly managed type 2 diabetes mellitus (T2DM) are at a higher risk of fractures or osteoporosis with deteriorated bone quality, even though their bone density is maintained [9]. In addition, the risk of fracture in patients with T2DM has recently received attention; the risk of fracture of the proximal femur in patients with T2DM has been shown to be a significant risk factor, approximately 1.8 times higher in men and 2.1 times higher in women [10].

On the other hand, skeletal muscle mass is known to decrease with age, and the age-related decline in skeletal muscle mass is one of the indicators used to assess sarcopenia, along with grip strength and walking speed [11]. Older patients with sarcopenia have been reported to have decreased activities of daily living (ADL) and more fractures due to falls [12], and identifying the factors that influence the decline in skeletal muscle mass is crucial for maintaining ADL in older people. Patients with T2DM are known to have a higher incidence of sarcopenia compared to non-T2DM patients [13].

Osteoporosis and sarcopenia are thought to be closely related and likely to coexist because they share many common factors, such as age-related declines in sex hormones, vitamin D deficiency, and reduced mechanical load [3]. Although a relationship between osteoporosis and sarcopenia has recently been reported, the relationship between the bone and muscle in patients with T2DM remains unclear. In this cross-sectional study, therefore, we investigated the relationship between bone metabolism markers and bone mineral density with sarcopenia in T2DM patients.

## 2. Methods

### 2.1. Research Subjects

This cross-sectional study was part of the ongoing prospective cohort study known as the KAMOGAWA-DM cohort study [14]. The local research ethics committee permitted this study (No. RBMR-E-466-6 and ERB-C-774-2). All patients provided informed consent through written documentation.

The study included patients with T2DM who were undergoing treatment at the Department of Diabetes Medicine at Kameoka City Hospital. These patients underwent measurements of bone metabolism markers, including bone alkaline phosphatase (BAP) and tartrate-resistant acid phosphatase 5b (TRACP-5b), bone mineral density measurements using dual-energy X-ray absorptiometry (DEXA, Lunar Prodigy, GE Healthcare, Madison, WI, USA), body composition measurements using bioelectrical impedance analysis (BIA, InBody 770, InBody Japan, Tokyo, Japan), and handgrip strength measurements. TRACP-5b was measured as a bone resorption marker. TRACP-5b was selected because of its high analytical stability and its minimal influence by renal function, making it a reliable indicator of bone resorption in patients with varying degrees of renal impairment [15]. Patients who were taking steroids or osteoporosis treatment drugs, those with rheumatoid arthritis or active malignancies, and cases where data were duplicated or insufficient were excluded from the study. There were no patients with hyperthyroidism, Cushing’s syndrome, or hypogonadism.

### 2.2. Data Collection and Definition of Osteoporosis and Sarcopenia

Data on duration of diabetes, exercise habits, smoking status, and family history of diabetes were collected using a standardized questionnaire. Based on the questionnaire results, the participants were categorized as current smokers or not, and as exercisers or not. In this study, we conducted a questionnaire among the participants, asking them if they performed some kind of exercise at least once a week or not, and we defined the participants who performed some kind of exercise at least once a week as exercisers. Medication data, including medications for diabetes and osteoporosis treatment, were collected from medical records. In addition, venous blood samples were collected after an overnight fast to measure creatinine (Cr), hemoglobin A1c (HbA1c), BAP, and TRACP-5b levels. Estimated glomerular filtration rate (eGFR) was calculated using the following equation: eGFR = 194 × Cr − 1.094 × age − 0.287 (mL/min/1.73 m^2^) (× 0.739 for women) [16].

Bone mineral density (BMD) (g/cm^2^) of the femur, femoral neck, and lumbar spine (L2–L4) was assessed using the DEXA method. Young adult mean (YAM) percentages and T-scores, the number of standard deviations (SDs) between the mean BMD of the participant and the mean of the reference population matched for sex and race [17], were automatically analyzed. We have classified skeletal status based on the WHO criteria using T-scores, where a T-score ≥ −1.0 was defined as normal, −2.5 < T-score < −1.0 as osteopenia, and a T-score ≤ −2.5 as osteoporosis [18]. Trabecular bone score (TBS) on spine images (using DEXA data from L1–L4) was evaluated using TBS Insight 2.2 software (Med-Imaps, Pessac, France) [19]. We calculated the prevalence of abnormal TBS using a cutoff value of less than 1.230 [20].

Skeletal muscle mass index (SMI) (kg/m^2^) was calculated as appendicular muscle mass (kg), assessed by the BIA method, divide height squared (m^2^) [21]. SMI of less than 5.7 kg/m^2^ in women and 7.0 kg/m^2^ in men was defined as low muscle mass. Handgrip strength of less than 18 kg in women and 28 kg in men was defined as low handgrip strength (low muscle strength). Sarcopenia was defined as having both low muscle mass and low grip strength [22]. Physical function, such as a 6-min walking test or a five-chair-standing test, was not assessed.

### 2.3. Statistical Analysis

The data were presented as means (SD) or frequencies of potential confounding variables.

The correlation between SMI or handgrip strength and bone metabolism markers and BMD or TBS was examined using the Spearman rank correlation coefficient. Differences in TRACP-5b concentrations or BMD of femoral neck with and without low handgrip strength, low muscle mass, and sarcopenia were tested by the Mann–Whitney U test. Logistic regression analysis was used to evaluate the relationship of TRACP-5b or BMD of the femoral neck to the presence of low handgrip strength, low muscle mass, and sarcopenia, adjusting for age, sex, exercise, and smoking. Receiver operator characteristic (ROC) analyses were performed to calculate the area under the ROC curve (AUC) of the ability of TRACP 5b or BMD of femoral neck for the presence of low handgrip strength, low muscle mass, and sarcopenia.

The statistical analyses were performed using the JMP version 13.2.1 software (SAS Institute Inc., Cary, NC, USA), and a *p*-value <0.05 was considered statistically significant.

## 3. Results

**Figure 1:**
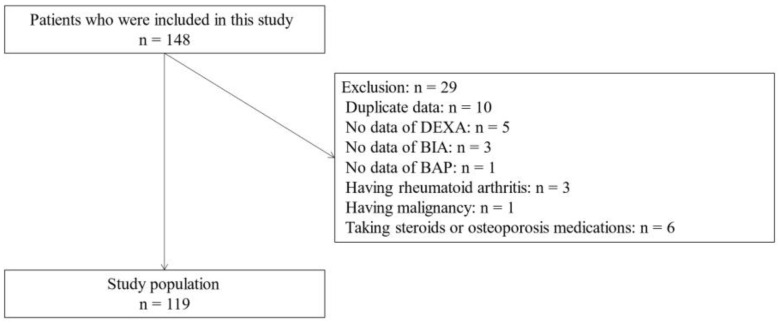
Inclusion and exclusion flow. DEXA, dual-energy X-ray absorptiometry; BIA, bioelectrical impedance analysis; BAP, bone alkaline phosphatase.

This cross-sectional study included 148 people with type 2 diabetes (Figure 1). Among them, 10 patients’ data were duplicates, 5 patients did not receive DEXA, 3 patients did not receive BIA, one patient did not receive BAP measurement, 3 patients had rheumatoid arthritis, 1 patient had malignancy, and 6 patients were already taking steroids or osteoporosis medications. Finally, data from 119 patients were used for this study.

Table 1 presents the clinical characteristics of the current study subjects. Among 119 subjects, 76 were women (mean age 70.8 ± 9.1 years, BMI 25.0 ± 4.5 kg/m^2^, duration of diabetes 11.6 ± 9.1 years) and 43 were men (mean age 75.0 ± 4.9 years, BMI 22.9 ± 3.4 kg/m^2^, duration of diabetes 17.9 ± 11.1 years). Mean lumbar spine, femur, and femoral neck BMD, SMI, and handgrip strength were 0.89 ± 0.19 g/cm^2^, 0.74 ± 0.13 g/cm^2^, 0.59 ± 0.11 g/cm^2^, 6.2 ± 0.9 kg/m^2^, and 21.6 ± 5.3 kg in women, respectively. For men, the corresponding values were 1.05 ± 0.21 g/cm^2^, 0.88 ± 0.18 g/cm^2^, 0.73 ± 0.17 g/cm^2^, 7.1 ± 0.9 kg/m^2^, and 32.3 ± 7.2 kg, in the same order. Overall, TBS, BAP, and TRACP-5b averaged 1.33 ± 0.10, 15.4 ± 7.0 U/L, and 488.1 ± 196.4 mU/dL, respectively. A total of 26 subjects (21.8%) had low handgrip strength, 45 (37.8%) had low muscle mass, and 18 (15.1%) had sarcopenia.

Table 2 presents the relationship between bone metabolism markers and handgrip strength or SMI. Significant correlations were observed between handgrip strength or SMI and TRACP-5b in both sexes (correlation coefficients were −0.50 (*p* < 0.001) in handgrip strength, −0.41 (*p* = 0.007) in SMI in men; correlation coefficients were −0.25 (*p* = 0.032) in handgrip strength, −0.21 (*p* = 0.073) in SMI in women). Furthermore, significant correlations were observed between BMD of the whole neck or femoral neck and handgrip strength or SMI in both sexes.

Table 3 presents the relationship between TBS and handgrip strength or SMI. No significant associations were observed between handgrip strength or SMI and TBS in either sex (correlation coefficients were 0.24 (*p* = 0.113) in handgrip strength, −0.001 (*p* = 0.995) in SMI in men; correlation coefficients were 0.11 (*p* = 0.340) in handgrip strength, 0.09 (*p* = 0.459) in SMI in women).

**Figure 2:**
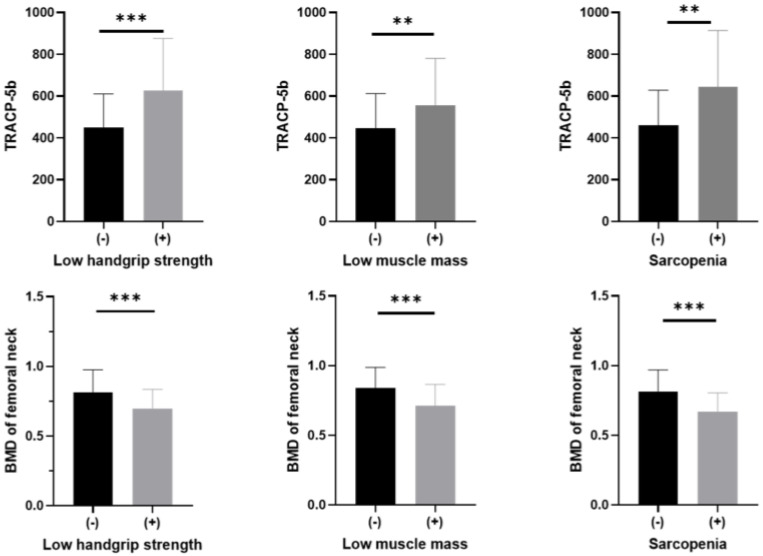
The concentrations of TRACP-5b or BMD of femoral neck with and without low handgrip strength, low muscle mass, and sarcopenia. Differences in TRACP-5b concentrations or BMD of femoral neck with and without low handgrip strength, low muscle mass, and sarcopenia were tested by the Mann–Whitney U test. ***, *p* < 0.001; **, *p* < 0.01. BMD, bone mineral density; TRACP-5b, tartrate-resistant acid phosphatase 5b.

The concentrations of TRACP-5b with low handgrip strength (624.9 ± 250.9 vs. 449.8 ± 160.0 mU/dL, *p* < 0.001), low muscle mass (560.6 ± 223.2 vs. 440.0 ± 162.1 mU/dL, *p* < 0.001), and sarcopenia (643.8 ± 261.9 vs. 455.7 ± 165.6 mU/dL, *p* < 0.001) were significantly higher compared to those without and BMD of femoral neck with low handgrip strength (0.56 ± 0.12 vs. 0.67 ± 0.16 g/cm^2^, *p* = 0.002), low muscle mass (0.58 ± 0.14 vs. 0.68 ± 0.16 g/cm^2^, *p* = 0.001), and sarcopenia (0.54 ± 0.12 vs. 0.66 ± 0.16 g/cm^2^, *p* = 0.002) were significantly lower compared to those without (Figure 2).

Table 4 presents the results of logistic regression analysis regarding the relationship between TRACP-5b or BMD of the femoral neck and low handgrip strength, low SMI, and the presence of sarcopenia. Even after adjustment for confounders, TRACP-5b was associated with the presence of low handgrip strength (odds ratio (OR) of Δ 10 incremental 1.04, 95% confidence interval (CI) 1.01–1.08, *p* = 0.008), low SMI (OR of Δ 10 incremental 1.03, 95% CI 1.00–1.05, *p* = 0. 008), and sarcopenia (OR of Δ 10 incremental 1.05, 95% CI 1.01–1.10, *p* = 0.008). Similarly, BMD of the femoral neck was associated with the presence of low handgrip strength (OR of Δ 10 incremental 0.48, 95% CI 0.28–0.84, *p* = 0.010), low SMI (OR of Δ 10 incremental 0.50, 95% CI 0.34–0.75, *p* < 0.001), and sarcopenia (OR of Δ 10 incremental 0.30, 95% CI 0.14–0. 68, *p* = 0.004).

**Figure 3:**
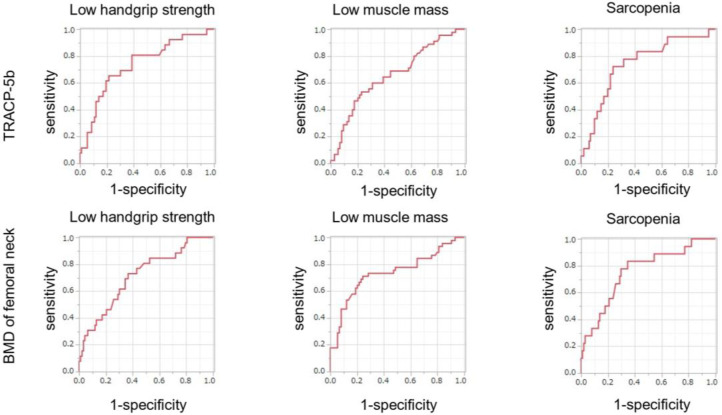
Receiver operating characteristic (ROC) analyses describing the predictive value of TRACP 5b or BMD of femoral neck for the presence of low handgrip strength, low muscle mass, and sarcopenia. Receiver operating characteristic curve and area under the ROC curve (AUC) showing the ability of TRACP 5b or BMD of femoral neck for the presence of low handgrip strength, low muscle mass, and sarcopenia. The optimal cutoff point of TRACP-5b for the presence of low handgrip strength, low muscle mass, and sarcopenia were 560 (AUC 0.74 [95% CI 0.61–0.83], sensitivity = 0.65, specificity = 0.78, *p* < 0.001), 519 (AUC 0.66 [95% CI 0.55–0.75], sensitivity = 0.53, specificity = 0.77, *p* = 0.002), and 560 (AUC 0.74 [95% CI 0.60–0.85], sensitivity = 0.72, specificity = 0.76, *p* < 0.001), respectively. The optimal cutoff point of BMD of femoral neck for the presence of low handgrip strength, low muscle mass, and sarcopenia were 0.75 (AUC 0.71 [95% CI 0.59–0.81], sensitivity = 0.73, specificity = 0.67, *p* < 0.001), 0.74 (AUC 0.74 [95% CI 0.63–0.82], sensitivity = 0.71, specificity = 0.76, *p* < 0.001), and 0.74 (AUC 0.76 [95% CI 0.61–0.86], sensitivity = 0.83, specificity = 0.65, *p* < 0.001), respectively. BMD, bone mineral density; TRACP-5b, tartrate-resistant acid phosphatase 5b.

Figure 3 depicts the receiver operating characteristic (ROC) curve of the correlation between TRACP-5b or BMD of femoral neck and the presence of sarcopenia. According to the ROC curve, TRACP-5b or BMD of femoral neck was a good marker for sarcopenia at a cutoff value of 560 mU/dL or 0.74 g/cm^2^.

## 4. Discussion

In this study, we investigated the relationship of bone metabolic markers or bone mineral density (BMD) to the presence of sarcopenia in patients with T2DM. As a result, significant correlations were observed between handgrip strength or SMI and TRACP-5b or BMD of femoral neck in both men and women, and an association between TRACP-5b or BMD of femoral neck and sarcopenia in patients with T2DM.

In a previous study by Hida et al., it was reported that the group with fragility fractures had significantly lower muscle mass and a higher rate of sarcopenia [23], and another study by Yoo et al., also reported a higher prevalence of sarcopenia in patients with proximal femur fractures [24], and there are numerous reports of a correlation between bone density and muscle mass. This may be related to the fact that there are factors that act in common between osteoporosis and sarcopenia, such as myokine, a cytokine secreted by muscle, and sclerostin, secreted by bone cells.

The possible mechanisms between TRACP-5b and sarcopenia are below. TRACP-5b is an enzyme found only in osteoclasts and is known as a bone resorption marker because it leaks into the blood with increased bone resorption, and receptor activator of nuclear factor-kappa B ligand (RANKL) expression is closely related to TRACP-5b [25]. Irisin, a myokine, inhibits bone resorption via activation of osteoblasts and suppression of RANKL expression [26], and its secretion is increased by exercise. Irisin secretion has been reported to be decreased in patients with T2DM [27]. Irisin is known to have an inhibitory effect on sarcopenia [28]. Thus, although we did not measure the concentration of irisin and RANKL, low irisin concentration, which has a close association with sarcopenia, might be related to the higher concentration of TRACP-5b.

Another possible mechanism is below. Lack of exercise, which is known to be a risk factor for sarcopenia, can cause osteoporosis. It has been reported that bone mass is reduced in patients who lie in bed for long periods of time and in astronauts, and mechanical loading is important for the maintenance of bone mass. Osteocytes are responsible for sensing mechanical loading in bone tissue, and sclerostin, secreted by osteocytes, inhibits bone formation by suppressing Wnt signaling. Although sclerostin is not a direct bone resorption marker like TRACP-5b, its function as a negative regulator of bone formation is well established. The secretion of sclerostin has been reported to be suppressed by mechanical loading [29], which may also suggest that osteocytes play a role in mechanosensing. Increased sclerostin levels have been reported in patients with type 2 diabetes mellitus (T2DM) [30], and serum sclerostin concentration has been shown to be negatively correlated with skeletal muscle mass [31]. In this study, serum sclerostin levels were not measured; however, higher sclerostin concentrations, which are known to be associated with sarcopenia, may be indirectly related to the elevated TRACP-5b levels observed in our participants. Nevertheless, given the cross-sectional design, causality cannot be inferred from these associations.

The fragility of cortical bone morphology is known to be an important factor for bone quality. According to reports using hip structural analysis (HSA) and peripheral quantitative computed tomography (pQCT), patients with DM have thinner cortical bone [32]. In addition, high-resolution pQCT (HR-pQCT) showed that cortical bone was more porous in the DM group than in the non-DM group. Cortical bone porosity has been reported to be an important factor affecting bone strength independently of bone mineral density [33], and a study in patients with T2DM reported increased cortical bone porosity in patients with fragility fractures [34,35]. In this study, only BMD of the femur, not BMD of the lumbar spine, was associated with sarcopenia. The femur is known to have more cortical bone than the lumbar spine, suggesting that it may have suffered more bone quality loss due to DM than the lumbar spine. There is an association between cortical bone fragility and muscle mass, as it has been reported that cortical bone area and thickness were reduced in patients with sarcopenia [36].

TBS is considered a bone quality assessment index that reflects trabecular microarchitecture, which is different from BMD. In patients with T2DM, it is known that bone quality is often impaired even when BMD is preserved or elevated [37]. Therefore, TBS is thought to be useful as an index that can evaluate fracture risk not captured by BMD [38]. In the present study, we did not observe any association between TBS and handgrip strength or SMI in both men and women. Since TBS is currently limited to the assessment of the lumbar spine and does not evaluate bone quality at other skeletal sites, such as the femur, it is possible that the relationship between bone quality and muscle function was not fully assessed. Thus, further studies are needed to clarify these associations.

This study has several notable limitations. Firstly, it is a cross-sectional study. Secondly, this study defines sarcopenia based solely on handgrip strength and muscle mass, without evaluating physical function; based on the AWGS2019, low muscle mass and low physical function can be diagnosed as sarcopenia without meeting low grip strength, which may underestimate the number of individuals with sarcopenia. Thirdly, this study adjusted for age, gender, exercise habits, and smoking as confounding factors, but did not take into account dietary habits or sex hormone levels. Fourthly, because of a lack of a sufficient number of cases, we have not included antidiabetic drugs, which might affect bone/muscle relationships, as covariates. Fifthly, no priori sample size calculation or statistical power analysis was conducted, which may limit the reliability of the observed associations. Further studies are needed to more accurately examine the relationship between osteoporosis and sarcopenia in T2DM patients.

In conclusion, bone metabolic markers were associated with sarcopenia in patients with T2DM.

## Figures and Tables

**Table 1:** Clinical characteristics of study participants.

n	119
Age (years)	72.3 ± 8.1
Sex (men/women)	43 (36.1)/76 (63.9)
Duration of diabetes (years)	13.8 ± 10.2
Height (cm)	157.1 ± 8.9
Body weight (kg)	59.9 ± 11.8
Body mass index (kg/m^2^)	24.2 ± 4.2
Biguanide (yes)	39 (32.8)
Thiazolidine (yes)	2 (1.7)
Sulfonylurea (yes)	21 (17.6)
Glinide (yes)	8 (6.7)
DPP4 inhibitor (yes)	46 (38.7)
SGLT2 inhibitor (yes)	16 (13.4)
α glucosidase inhibitor (yes)	17 (14.3)
GLP-1 receptor agonist (yes)	40 (33.6)
Insulin (yes)	38 (31.9)
Exercise (yes)	51 (42.9)
Current smoker (yes)	13 (10.9)
HbA1c (%)	7.4 ± 1.1
Creatinine (umol/L)	70.9 ± 25.5
Estimated glomerular filtration rate (mL/min/1.73m^2^)	65.5 ± 17.7
Trabecular bone score (n = 118)	1.33 ± 0.10
Abnormal TBS	19 (16.1)
Overlap of abnormal TBS with low BMD	18 (15.3)
Overlap of abnormal TBS with sarcopenia	3 (2.5)
Bone mineral density of the lumbar spine (g/cm^2^)	0.95 ± 0.22
Normal	74 (62.2)
Osteopenia	29 (24.4)
Osteoporosis	16 (13.4)
Bone mineral density of the whole neck (g/cm^2^)	0.79 ± 0.16
Normal	60 (50.4)
Osteopenia	40 (33.6)
Osteoporosis	19 (16.0)
Bone mineral density of the femoral neck (g/cm^2^)	0.64 ± 0.16
Normal	40 (33.6)
Osteopenia	34 (28.6)
Osteoporosis	45 (37.8)
Bone-specific alkaline phosphatase (U/L)	15.4 ± 7.0
Tartrate-resistant acid phosphatase 5b (mU/dL)	488.1 ± 196.4
Handgrip strength (kg)	25.5 ± 8.0
Low handgrip strength (yes)	26 (21.8)
Skeletal muscle mass index (kg/m^2^)	6.5 ± 1.0
Low muscle mass (yes)	45 (37.8)
Sarcopenia (yes)	18 (15.1)
Prior fragility fractures (yes)	91 (76.5)

Data are shown as number (percentage) or mean ± standard deviation.

**Table 2:** Association between osteoporosis marker and handgrip strength or SMI.

**Men**	**Handgrip Strength**		**SMI**	
	***r***	***p***	***r***	***p***
Bone-specific alkaline phosphatase	−0.31	0.040	−0.38	0.012
Tartrate-resistant acid phosphatase 5b	−0.50	<0.001	−0.41	0.007
BMD of the lumbar spine	0.16	0.311	0.15	0.348
BMD of whole neck	0.36	0.018	0.44	0.003
BMD of femoral neck	0.39	0.011	0.35	0.020
**Women**	**Handgrip strength**		**SMI**	
	***r***	***p***	***r***	***p***
Bone-specific alkaline phosphatase	−0.02	0.848	−0.08	0.519
Tartrate-resistant acid phosphatase 5b	−0.25	0.032	−0.21	0.073
BMD of the lumbar spine	0.09	0.463	0.39	<0.001
BMD of whole neck	0.37	0.001	0.59	<0.001
BMD of femoral neck	0.34	0.003	0.50	<0.001

Spearman’s rank correlation coefficient is performed. BMD, bone mineral density; SMI, Skeletal muscle mass index.

**Table 3:** Association between TBS and handgrip strength or SMI.

**Men**	**Handgrip Strength**		**SMI**	
	***r***	***p***	***r***	***p***
Trabecular bone score	0.24	0.113	−0.001	0.995
**Women**	**Handgrip strength**		**SMI**	
	***r***	***p***	***r***	***p***
Trabecular bone score	0.11	0.340	0.09	0.459

Spearman’s rank correlation coefficient is performed.

**Table 4:** Logistic regression analyses of osteoporosis markers for low handgrip strength, low SMI, and sarcopenia.

	**Low Handgrip Strength**		**Low SMI**		**Sarcopenia**	
	**OR (95% CI)**	***p***	**OR (95% CI)**	***p***	**OR (95% CI)**	***p***
Age (year)	1.26 (1.12–1.41)	<0.001	1.12 (1.05–1.20)	<0.001	1.34 (1.13–1.58)	<0.001
Men	1.85 (0.55–6.20)	0.321	1.46 (0.58–3.63)	0.421	6.03 (1.30–28.1)	0.021
Exercise	0.66 (0.20–2.19)	0.495	1.39 (0.57–3.37)	0.463	0.49 (0.11–2.13)	0.339
Smoking	0.46 (0.04–4.50)	0.508	1.54 (0.42–5.70)	0.519	0.95 (0.09–10.3)	0.964
TRACP-5b (per 10 mU/dL incremental)	1.04 (1.01–1.08)	0.008	1.03 (1.00–1.05)	0.008	1.05 (1.01–1.10)	0.008
	**Low Handgrip strength**		**Low SMI**		**Sarcopenia**	
	**OR (95% CI)**	***p***	**OR (95% CI)**	***p***	**OR (95% CI)**	***p***
Age (year)	1.29 (1.14–1.47)	<0.001	1.13 (1.05–1.22)	<0.001	1.47 (1.18–1.84)	<0.001
Men	3.70 (0.90–15.2)	0.069	3.55 (1.15–11.0)	0.028	26.8 (3.22–223)	0.002
Exercise	1.07 (0.31–3.64)	0.913	1.97 (0.77–5.04)	0.159	1.59 (0.29–8.88)	0.595
Smoking	0.58 (0.06–5.51)	0.633	1.56 (0.42–5.79)	0.505	1.45 (0.12–17.3)	0.771
BMD of femoral neck (per 10 g/cm^2^ incremental)	0.48 (0.28–0.84)	0.010	0.50 (0.34–0.75)	<0.001	0.30 (0.14–0.68)	0.004

TRACP-5b, tartrate-resistant acid phosphatase 5b; BMD, bone mineral density; SMI, skeletal muscle mass index.

## Data Availability

The data that support the findings of this study are available from the corresponding author, YH, upon reasonable request.
